# Methodological challenges in studies of the role of blood lipids variability in the incidence of cardiovascular disease

**DOI:** 10.1186/s12944-021-01477-x

**Published:** 2021-05-19

**Authors:** Leonelo E. Bautista, Oscar L. Rueda-Ochoa

**Affiliations:** 1 Department of Population Health Sciences, School of Medicine and Public Health, University of Wisconsin-Madison, Madison, USA; 2 Department of Basic Sciences, Director Cardiovascular Research Group, Universidad Industrial de Santander, Bucaramanga, Colombia

It has long been known that high levels of total and LDL-cholesterol, and low levels of HDL increase the risk of developing atherosclerotic cardiovascular diseases [1]. Recent studies suggest that high intra-individual variability in blood lipids may increase the risk of major cardiovascular events (MACE: non-fatal myocardial infarction, stroke, or cardiovascular death), independently of individual average levels [2]. Here we discuss some methodological challenges in studies of the causal effect of blood lipids variability (BLV) on the risk of MACE, and suggest possible solutions.

Studies of the BLV-MACE association have shown that high intra-individual blood lipids variability (BLV) is independently associated with MACE in different types of patients [3–7], as well as in the general population [8, 9].Visit to visit BLV has been measured by conducting serial measurements of blood lipids and calculating their measure-to-measure variability as the standard deviation (SD), the coefficient of variation (CV), the average successive variability (ASV = ∑(*X*_*i*_ *– X*_*i + 1*_)/n) or the variance Independent of the mean (VIM = (*k* SD/ ${\overline{x}}^p$), where *k = M*^*p*^, and *p* is the regression coefficient of log (SD) over the log($\overline{X}\Big)$ [9, 10]. These estimates of individual BLV are then included in regression models to estimate the between-subject effect of BLV. Measures such as SD and CV are strongly related to the mean of visit values, while VIM is independent of the mean [2].

An accurate assessment of individual BLV requires multiple measurements at given intervals of time. Unfortunately, there is considerable uncertainty about how many measurements are needed and how long the intervals should be in order to get an assessment of BLV that is accurate and also etiologically relevant. Researchers should not assume that because data are available, BLV based on that data is accurate or biologically and clinically relevant. Average lipid levels, at a given point in time, reflects accumulated exposure up to that time, and is unequivocally related to the risk of MACE [1]. In contrast, it is uncertain whether BLV measured over a period of weeks or months accurately reflects a cumulative exposure, particularly in patients receiving cholesterol-lowering drugs. This uncertainty is due in part to our limited knowledge on BLV over time. In a meta-analysis conducted by Smith et al. [11] the coefficient of variation (CV = standard deviation/mean) for HDL were 5.5, 5.8, and 7.8% when measurements were conducted daily, weekly, and monthly, respectively. Corresponding values for LDL were 6.1, 6.2, and 10.8%. Similar findings have been reported in more recent studies [12, 13]. These studies seem to indicate BLV is low, at least when measured in a relative scale. On the other hand, studies on age-related BLV indicate that LDL cholesterol levels increase with age and reach a plateau in men between the age of 50 and 60 years, and in women between the age of 60 and 70 years [14], while HDL cholesterol levels diminish or have minimal changes with age [15, 16]. Taken together, these studies suggest BLV measured in intervals of even years may be small and of little clinical significance, after accounting for age-related changes. Therefore, well-designed studies describing the accuracy of BLV patterns are highly desirable. Knowledge generated in those studies would inform the design and interpretation of future studies, identify high-risk individuals, and determine what measurement intervals and indicators of BLV are clinically relevant.

Evidence of mechanisms explaining a BLV-MACE association is limited. One proposed hypothesis is that high BLV promotes the development and progression of atherosclerotic plaques. Clark et al. showed that BLV was associated with progression of coronary atherosclerotic plaques, measured as the proportion of the arterial wall occupied by the plaque, in patients included in trials assessing the effect of intensive lipid lowering with statins [17]. However, the BLV effects on plaque development were weak, were not present in individuals who achieved treatment target, and were not independent of average levels of blood lipids. On the other hand, in patients receiving cholesterol-lowering drugs, the BLV-MACE association may also be just a consequence of poor treatment adherence [18].

The BLV-MACE association observed in several studies, though statistically significant, has been consistently weak. The average increases in risks of MACE associated with LDL-variability ranged between 10 and 34% in studies in cardiovascular patients [3–6], and between 6 and 11% in the general population [8, 9]. Associations from studies in treated patients should be cautiously interpreted. Interventions to reduce the risk of MACE in these patients are guided by their levels of cardiovascular risk factors. If those interventions are more frequent or intense in patients with high levels of blood lipids, the risk of MACE in patients with high BLV may be underestimated, since BLV increases with average blood lipid levels. This implies that data on co-interventions must be collected at the times blood lipids are measured, to control for their confounding effect. On the other hand, the effect of BLV from studies in the general population are too weak (maximum hazard ratio of 1.11) and could be explained by a confounding factor that increases the proportion of individuals with high BLV and the risk of MACE by just 46% [19].

It is also uncertain whether BLV is a modifiable therapeutic target, beyond the modification of average levels, and how this may be achieved. Moreover, before implementation of BLV measurement in the clinical context it is necessary to ascertain whether different measures of BLV improve predictability of MACE to the same degree, and which may be easier to use in patient care. Implementation is also hampered by the fact that BLV measures, such as SD, CV, and VIM are study-population specific and, therefore, their values cannot be applied to other populations. Moreover, the BLV-MACE association observed in several studies, though statistically significant, may not improve the performance of models excluding BLV or predictions from a cardiovascular risk score [20].

On the other hand, in most studies, BLV has been evaluated through short-time intervals and taken as the baseline exposure. This implies, without due justification, that BLV is a time-fixed variable that does not change or changes little over the follow-up period. Longitudinal follow-up studies, with repeated evaluations of BLV, time-dependent risk factors, and MACE or surrogates of MACE, such as carotid artery intima-media thickness and plaque stability, could be used to test this assumption and elucidate both the stability of BLV and its within-individual effects. In addition, Cox proportional hazard model has been used to assess the BLV-MACE association in most studies [3, 5–9, 21]. Although appropriate for the analysis of survival data, Cox regression does not take advantage of the correlation across the repeated measurements of blood lipids or the observed trajectory in average lipid levels. Moreover, Cox regression only considers between group variability, leaving out the estimation of within-individual and total effects of BLV. Other analytical approaches, such as multilevel/mixed linear regression models [22], joint modelling [23, 24], and g methods [25, 26], could be helpful to address the limitations of Cox regression. In particular, these methods could be useful to estimate the potential impact of pharmacological interventions to reduce BLV using data from observational studies.

## Data Availability

Not applicable.

## References

[1] Grundy Scott M, Stone Neil J, Bailey Alison L, Beam C, Birtcher Kim K, Blumenthal Roger S, Braun Lynne T, de Ferranti S, Faiella-Tommasino J, Forman Daniel E, Goldberg R, Heidenreich Paul A, Hlatky Mark A, Jones Daniel W, Lloyd-Jones D, Lopez-Pajares N, Ndumele Chiadi E, Orringer Carl E, Peralta Carmen A, Saseen Joseph J, Smith Sidney C, Sperling L, Virani Salim S, Yeboah J (2018). 2018 aha/acc/aacvpr/aapa/abc/acpm/ada/ags/apha/aspc/nla/pcna guideline on the management of blood cholesterol. Circulation.

[2] Simpson WG (2019). Biomarker variability and cardiovascular disease residual risk. Curr Opin Cardiol.

[3] Waters DD, Bangalore S, Fayyad R, DeMicco DA, Laskey R, Melamed S, Barter PJ (2018). Visit-to-visit variability of lipid measurements as predictors of cardiovascular events. J Clin Lipidol.

[4] Bangalore S, Breazna A, DeMicco DA, Wun C-C, Messerli Franz H, null n (2015). Visit-to-visit low-density lipoprotein cholesterol variability and risk of cardiovascular outcomes. J Am Coll Cardiol.

[5] Lee EY, Yang Y, Kim H-S, Cho J-H, Yoon K-H, Chung WS, Lee S-H, Chang K (2018). Effect of visit-to-visit ldl-, hdl-, and non-hdl-cholesterol variability on mortality and cardiovascular outcomes after percutaneous coronary intervention. Atherosclerosis..

[6] Boey E, Gay GMW, Poh K-K, Yeo T-C, Tan H-C, Lee C-H (2016). Visit-to-visit variability in ldl- and hdl-cholesterol is associated with adverse events after st-segment elevation myocardial infarction: a 5-year follow-up study. Atherosclerosis..

[7] Bangalore S, Fayyad R, Messerli FH, Laskey R, DeMicco DA, Kastelein JJP, Waters DD (2017). Relation of variability of low-density lipoprotein cholesterol and blood pressure to events in patients with previous myocardial infarction from the ideal trial. Am J Cardiol.

[8] Kim Mee K, Han K, Park Y-M, Kwon H-S, Kang G, Yoon K-H, Lee S-H (2018). Associations of variability in blood pressure, glucose and cholesterol concentrations, and body mass index with mortality and cardiovascular outcomes in the general population. Circulation..

[9] Kim MK, Han K, Kim HS, Park YM, Kwon HS, Yoon KH, Lee SH (2017). Cholesterol variability and the risk of mortality, myocardial infarction, and stroke: a nationwide population-based study. Eur Heart J.

[10] Rothwell PM, Howard SC, Dolan E, O'Brien E, Dobson JE, Dahlöf B, Sever PS, Poulter NR (2010). Prognostic significance of visit-to-visit variability, maximum systolic blood pressure, and episodic hypertension. Lancet..

[11] Smith SJ, Cooper GR, Myers GL, Sampson EJ (1993). Biological variability in concentrations of serum lipids: sources of variation among results from published studies and composite predicted values. Clin Chem.

[12] Rivera-Coll A, Fuentes-Arderiu X, Díez-Noguera A (1994). Circadian rhythmic variations in serum concentrations of clinically important lipids. Clin Chem.

[13] Marcovina SM, Gaur VP, Albers JJ (1994). Biological variability of cholesterol, triglyceride, low- and high-density lipoprotein cholesterol, lipoprotein(a), and apolipoproteins a-i and b. Clin Chem.

[14] Gobal FA, Mehta JL (2010). Management of dyslipidemia in the elderly population. Ther Adv Cardiovasc Dis.

[15] Wilson PWF, Anderson KM, Harri T, Kannel WB, Castelli WP (1994). Determinants of change in total cholesterol and hdl-c with age: the Framingham study. J Gerontol.

[16] Schubert CM, Rogers NL, Remsberg KE, Sun SS, Chumlea WC, Demerath EW, Czerwinski SA, Towne B, Siervogel RM (2006). Lipids, lipoproteins, lifestyle, adiposity and fat-free mass during middle age: the fels longitudinal study. Int J Obes.

[17] Clark D, Nicholls SJ, St. John J, Elshazly MB, Kapadia SR, Tuzcu EM, Nissen SE, Puri R (2018). Visit-to-visit cholesterol variability correlates with coronary atheroma progression and clinical outcomes. Eur Heart J.

[18] Mann DM, Glazer NL, Winter M, Paasche-Orlow MK, Muntner P, Shimbo D, Adams WG, Kressin NR, Zhang Y, Choi H, Cabral H (2013). A pilot study identifying statin nonadherence with visit-to-visit variability of low-density lipoprotein cholesterol. Am J Cardiol.

[19] VanderWeele TJ, Ding P (2017). Sensitivity analysis in observational research: introducing the e-value introducing the e-value. Ann Intern Med.

[20] Stevens SL, McManus RJ, Stevens RJ (2019). The utility of long-term blood pressure variability for cardiovascular risk prediction in primary care. J Hypertens.

[21] Han BH, Han K, Yoon KH, Kim Mee K, Lee SH (2020). Impact of mean and variability of high-density lipoprotein-cholesterol on the risk of myocardial infarction, stroke, and mortality in the general population. J Am Heart Assoc.

[22] Hruschka DJ, Kohrt BA, Worthman CM (2005). Estimating between- and within-individual variation in cortisol levels using multilevel models. Psychoneuroendocrinology..

[23] Papageorgiou G, Mauff K, Tomer A, Rizopoulos D (2019). An overview of joint modeling of time-to-event and longitudinal outcomes. Annu Rev Stat Appl.

[24] Rueda-Ochoa OL, Rojas LZ, Ahmad S, van Duijn C, Ikram MA, Deckers JW, Franco OH, Rizopoulos D, Kavousi M (2018). Impact of cumulative SBP and serious adverse events on efficacy of intensive blood pressure treatment. A randomized clinical trial. J Hypertens.

[25] Taubman SL, Robins JM, Mittleman MA, Hernán MA (2009). Intervening on risk factors for coronary heart disease: an application of the parametric g-formula. Int J Epidemiol.

[26] Naimi AI, Cole SR, Kennedy EH (2017). An introduction to g methods. Int J Epidemiol.

